# The Effect of Pretransplant Malignancy on the Outcome After Heart Transplantation: A Review

**DOI:** 10.3390/healthcare14213828

**Published:** 2026-11-09

**Authors:** Wilhelm Peter Mistiaen

**Affiliations:** Faculty of Medicine and Health Sciences, University of Antwerp, 2610 Antwerp, Belgium; wilhelm.mistiaen@uantwerpen.be

**Keywords:** pretransplant malignancy, heart transplant, survival, post-transplant malignancy

## Abstract

**Highlights:**

This review deals with the outcome of heart transplantation in cancer survivors with end-stage heart failure of any cause. The results of the last ten years were collected and reviewed with respect to the outcome in terms of survival, cancer recurrence and appearance of new cancers. Papers studying solid organ transplantation were also included, if data for heart transplantation were provided.

**What are the main findings?**

- In most series, survival beyond one year after heart transplant in cancer survivors seems comparable to that for patients without pretransplant malignancy.
- The risk for post-transplant malignancies, especially for skin cancer, is significantly higher in patients who had pretransplant malignancy; results after heart transplantation seem worse compared to abdominal organ transplant.

**What are the implications of the main findings?**

- Cancer survivors with end-stage heart failure still could undergo heart transplantation after adequate screening by oncologists.
- Post-transplant malignancy requires strict follow-up for patients at higher risk.

**Abstract:**

**Objectives:** The number of cancer survivors increases steadily over time because of improved cancer treatment. The number of survivors needing heart transplantation is also rising. This review aims to address the post-transplant survival and cancer incidence among patients with pretransplant malignancy. **Methods:** A literature search was performed in Web of Science and PubMed, for the last 10 years using the search terms: (heart OR cardiac) AND pretransplant* AND (malignancy OR cancer OR tumor OR neoplasm). Only full articles were included and assessed by the Newcastle–Ottawa Scale of their quality. **Results:** Nine manuscripts were identified. Only four articles analyzed patients undergoing exclusively heart transplantation. In the other five manuscripts, solid organ transplant series were investigated. The outcomes were rates of survival, cancer recurrence, or de novo malignancy. Four series were derived from one center, the other five were region or nationwide surveys. High-risk pretransplant malignancy affected long-term survival. The risk for post-transplant malignancy, especially for skin cancer, was higher in patients with pretransplant malignancy. Outcome in thoracic organ transplant was worse compared to abdominal organ transplant. Age, male gender, and white race were risk factors for post-transplant skin cancer. **Conclusions:** Pretransplant malignancy does not preclude heart transplantation, but patients with this condition should undergo thorough oncologic screening before and after HTX.

## 1. Introduction

Heart transplantation (HTX) remains the definitive therapy for patients with end-stage heart failure. This procedure increases survival and quality-of-life. One-year survival exceeds ninety percent because of improvement of surgical techniques, perioperative care and organ donor management. However, long-term survival did not improve over time because of coronary allograft vasculopathy, malignancy, renal failure, infection, diabetes, hypertension and the effects of chronic immunosuppression [1]. Heart transplantation is also an option for cancer survivors suffering from end-stage heart failure, because of chemotherapy. Anthracyclines have high activity against hematologic malignancies and solid tumors, but these agents are known to cause myocardial damage. The cumulative dose is considered as the most important risk factor for left ventricular (LV) dysfunction [2,3], but histopathologic changes have already been documented after administration of 240 mg/m^2^ of doxorubicin [4]. Mechanisms of cardiac dysfunction include damage to DNA and to mitochondria, which are abundantly present in cardiac myocytes. Risk factors for cardiac damage include age above 65 years, female gender, prior radiotherapy of the chest, hypertension, use of other chemotherapeutic agents such as trastuzumab and pre-existing heart disease [2]. The risk caused by tyrosine kinase inhibitors seems to be lower [5,6,7,8]. Immune checkpoint inhibitors enhance immune responses against tumor cells but can also trigger immune-related myocarditis, which can sometimes be fatal [9,10]. The cardiac side effects of chemotherapy are an important issue, since cardiovascular disease is the leading cause of noncancer mortality in long-term cancer survivors. Cancer-related exposures may contribute to long-term cardiovascular risk, which can remain dormant for years [5,11,12,13,14]. Cardiac magnetic resonance imaging of asymptomatic survivors with normal echocardiograms showed a lower LV ejection fraction [14]. Although cardiotoxicity was only recorded in 7% of survivors of childhood malignancy, mortality was high in symptomatic patients. Moreover, freedom from symptoms does not ensure normal heart function. Children with abnormal ventricular systolic function after cancer treatment have higher mortality risk compared to those with normal systolic function [15]. Acute heart failure is reversible with adequate treatment, while delayed-onset heart failure significantly reduces survival. Heart transplantation in patients with chemotherapy-induced heart failure can be performed with good 10-year survival [5]. This review addresses the effect of pretransplant malignancy (PTM) on post-transplant survival as primary endpoint, and cancer incidence (new and recurrent) as secondary endpoint.

## 2. Materials and Methods

**Figure 1:**
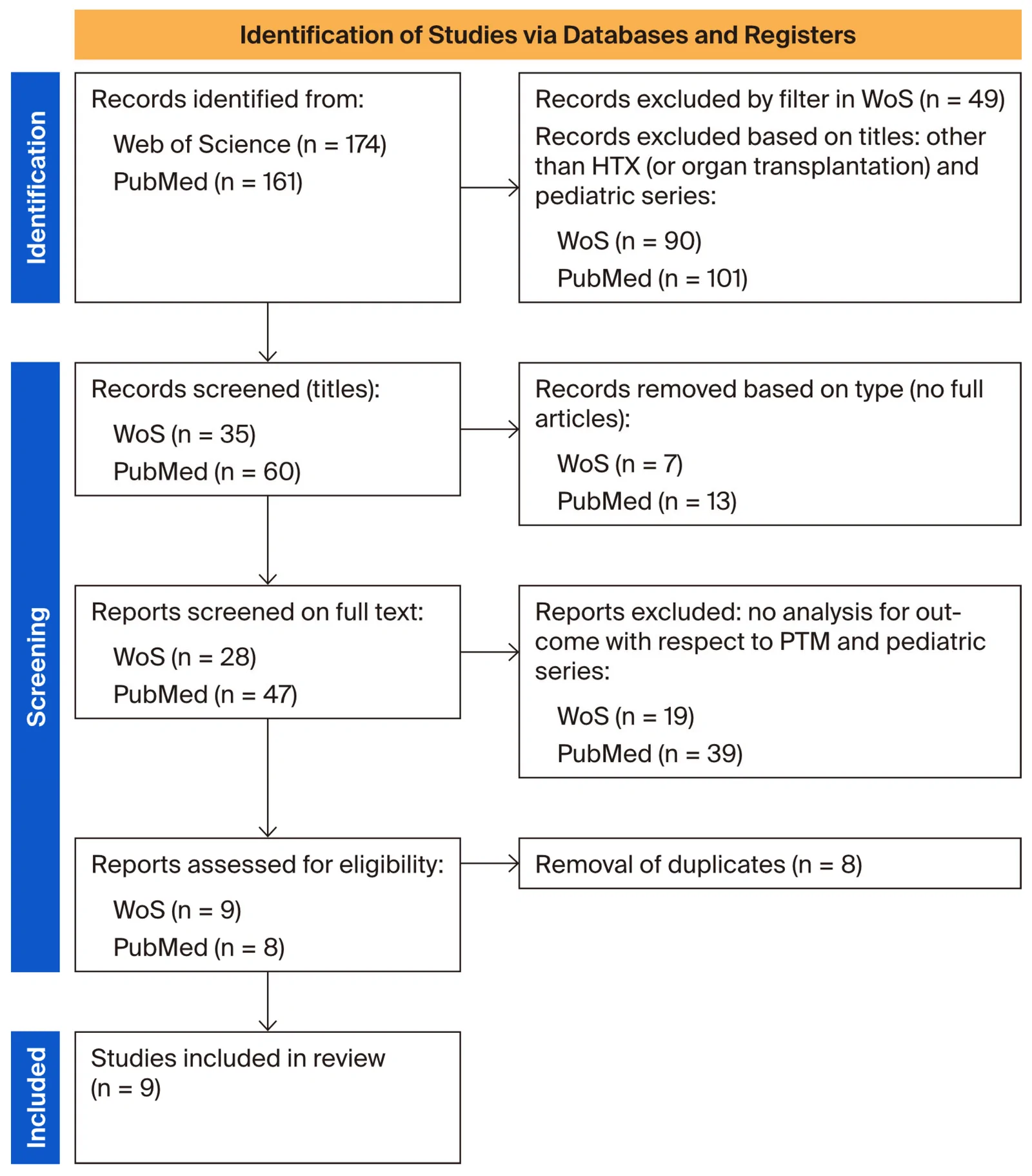
Search strategy.

A literature search was performed through the Web of Science and PubMed databases. There was no PROSPERO registration. Full compliance with PRISMA guidelines was not possible with only one author (Supplementary File S1). The search included the following strings of search terms: (heart OR cardiac) AND pretransplant* AND (malignancy OR cancer OR tumor OR neoplasm). To obtain the most recent advances, the inclusion was restricted from 1 January 2015 to 26 September 2026. Only papers in English were considered. Inclusion of papers was based on titles and on abstract. For Web of Science, the filter “articles” was used for “all databases”. This excluded most reviews, editorials, case reports, meeting abstracts and opinion papers. For PubMed, no field or filters (article type, article attributes or text availability) were used since almost all items were articles or reviews. Any remaining reviews, editorials and case reports from both sources were removed by title or abstract. Eligible articles included patients undergoing HTX, with versus without PTM and were based on medical records or databases. Articles dedicated to transplantation specifically other than HTX were also excluded. Both types of patients had to be drawn from the same cohort. The outcome (recurrent or new malignancy, survival) had to be presented as hazard ratio with 95% confidence intervals, cumulative incidence, or as percentages of post-transplant outcome for both patient groups. Series of solid organ transplants, which also included patients who underwent HTX, but were not analyzed separately, were grouped in another category, as comparison. The search strategy is depicted in Figure 1. The included articles were assessed for their quality by the Newcastle–Ottawa Scale. The main confounders for the assessment of quality included age, gender, race (or skin type). Because of the variety in study design and outcomes, a meta-analysis was precluded.

## 3. Results

### 3.1. Article Identification and Quality Control

In Web of Science, 174 items were identified. This was reduced to 125 after filtering for “articles”. In PubMed, 161 items were identified. After screening for title, abstract and full text, papers dealing with pediatric series and transplant explicitly other than HTX were excluded. This resulted finally in nine and eight papers, respectively, for the two sources. For the sake of reproducibility, duplicates were removed at the end of the process. Nine records were included for analysis [16,17,18,19,20,21,22,23,24], including 53,796 HTX-patients. Four papers studied solely HTX patients [17,22,23,24], one paper reported on heart and lung transplants [18], four series dealt with patients undergoing solid organ transplantation (SOTR), which also included HTX [16,19,20,21]. All papers can be classified as cohort studies and were subjected to the Newcastle–Ottawa Scale for their quality. Two papers did not reveal the specific number of patients with PTM but described the effect of PTM on outcome by calculating adjusted hazard ratios. Table 1 displays the quality assessment of the included series [16,17,18,19,20,21,22,23,24]. All papers can be considered of sufficient quality with respect to the criteria from the scale. Only one paper [19] reported a rate of missing patients during follow-up, at a rate of 11%. There was no reason to assume that this percentage would differ between patients with or without PTM. In the other manuscripts, this % was not stated. Median follow-up in these series varied between 3.5 and 8.6 years.

### 3.2. Baseline Patient Characteristics

Table 2 shows the included manuscripts [16,17,18,19,20,21,22,23,24] with mean patient ages, the number of HTX and solid organ transplantations (SOTR), the numbers of PTM, and the design. Two of the four papers [17,23], including solely HTX patients were derived from the united network organ sharing (UNOS) database, while two papers dealt with monocentric series. Of the four papers showing mixed series [16,19,20,21], only two provided more specific numbers for thoracic organ transplant [19] and for HTX [21], respectively. One of the included series dealt exclusively with the effect of sirolimus after transplantation [20]. The mean age of the included patients was consistently between 50 and 60 years. In almost all series, at least 60% of the patients were male.

### 3.3. Outcomes of the Included Series

Table 3 shows, from left to right the reference, the outcome under study, followed by the predictors (if available), the result, mostly presented as adjusted hazard ratio with the 95% confidence intervals, unless otherwise indicated. The obtained values are statistically significant if the range of the confidence interval excluded the value of one. In several series [17,22,23,24] the percentages were also provided. In one series, there were 11,434 patients undergoing SOTR and 1151 had PTM. In 443 patients, this malignancy was in remission. After exclusion of patients with cancer as indication for transplant, or incidental finding of cancer during transplantation, 10,716 patients were entered in a 2:1 propensity score match analysis. This resulted in a cohort of 886 patients without PTM versus 443 with PTM. For patients undergoing HTX, this was 58 versus 29 patients. Recipients with PTM had a decreased overall survival compared with recipients without PTM (10.3 years vs. 13.4 years). Recipients with PTM had a higher risk of cancer-specific mortality with a hazard ratio of 1.85; 95% (1.20–2.86) and of noncancer death with a hazard ration 1.29 (1.08–1.54). There was no separate risk analysis for patients undergoing HTX [16].

In one series [20] absolute numbers for post-transplant malignancies were presented. Four series were limited to one center, while five series were based on region or nationwide registries [17,19,21,23]. These registries showed generally less data about cancer stage, treatment and cancer free intervals.

The outcome after HTX with respect to mortality showed mixed results. An increased 1-year mortality in presence of PTM was observed, especially of the hematologic type, but there was no increase in the all-cause mortality between 1 and 5 years. However, cancer-related mortality between 1 and 5 years has increased significantly from 10.8% to 18.7% [17]. Another series [22] also showed no difference in 10-survival between patients with and without PTM, nor was there a difference in cancer-related mortality. One series showed a relatively large difference of 18.7% in 5-year survival between patients with and without PTM, but the absolute numbers were low, with only eight patients in the PTM group [24]. The outcome with respect to post-transplant malignancy showed a more consistent pattern: the presence of PTM increased the rate for most post-transplant malignancies [17,18,22,23]. This was especially true for post-transplant skin cancer of various types [18,21,22,23], and in patients of white race [22].

In series with mixed organ transplantation, a significant effect of PTM on all-cause and cancer-specific mortality was observed, especially with lung, digestive, melanoma and hematologic PTM [16]. Thoracic organ transplants showed in most series a worse outcome compared to abdominal transplants. The occurrence of skin cancer was 1.51 (1.26–1.82) times higher after thoracic organ transplant compared to abdominal organ transplant which had a reference value of 1.00. This effect was less important than the effect of age above 50 years with a hazard ratio of 2.65 (2.12–3.21) or being of white race with a hazard ratio of 7.79 (5.34–11.37) [19]. The 5-year cumulative incidence of keratinocyte carcinoma followed a comparable pattern (Supplementary File S1); for kidney and liver transplant, these numbers were respectively 11.20 (10.37–12.06) and 10.39 (9.12–11.75). For lung and heart transplants, these figures were higher, with respectively 14.67 (12.15–17.41) and 15.04 (12.32–18.02) [21]. The ‘novo cancer’ death rate for kidney transplant was 245/6516 (or 3.7%) and for liver transplant this was 93/2606 (or 3.6%). For HTX, this was 48/929 (5.2%) and for lung transplant, this was 20/705 (2.8%) [16]. The effects of age, gender and race were studied in some series, which can be seen in Table 3. The effects of comorbid conditions such as hypertension and chronic kidney disease were analyzed in only two series and had no significant effect [22,23]. Only diabetes showed an effect, but only on skin cancer in one series [23].

## 4. Discussion

### 4.1. Series Studying Solid Organ Transplantation

Mortality rate after SOTR was explored in only one series [16]. An increase in all-cause and cancer mortality was observed and overall survival decreased, from (10.3 years vs. 13.4 years). This excess of mortality was seen for all transplanted organs, but patients receiving thoracic organs had the highest risk. The excess mortality also persisted when patients with PTM were excluded. Remarkably, the observed increase in mortality was only significant with an interval between PTM and SOTR of at least 5 years, and not in patients with a shorter interval. More patients with high-risk PTM (lung cancer, hematologic malignancies, melanoma) received a transplant organ with a longer interval, which could offer a partial explanation. Cancer recurrence was higher with high-risk PTM, but there was also an increased risk for non-cancer death in these patients. A prolonged stay on the waiting list and the use of competing risk analysis in this series (which takes graft loss and non-cancer death into account) could also explain this observation [16]. The observed effect of the interval between cancer and transplantation contrasted with the results seen after more commonly performed cardiac operations, such as coronary artery bypass grafting [25] and surgical aortic valve replacement [26]. These investigations showed that a longer interval improved cancer-specific and overall survival.

Post-transplant rates of cancer were investigated in four series evaluating SOTR [18,19,20,21]. One series was dedicated exclusively to thoracic organ transplants and analyzed patients undergoing HTX separately [18]. Post-transplant skin cancer was the outcome under study, which is nearly five times the rate of all cancers combined in the overall US population. Improved survival due to newer immune suppressive agents and better screening techniques might contribute to this observation [19]. In most series, post-transplant malignancy rates were significantly increased in patients with PTM [18]. White race proved to be a major risk factor, while a dark skin had a protective effect. This could be related to the sun exposure: non-melanoma skin cancer occurred in 45% of the patients who underwent SOTR in Australia, compared to 10–15% in European countries [18]. Higher age and male gender were also risk factors but in a lower degree [18,19]. A transplantation of thoracic organs showed a higher risk for squamous cell carcinoma and skin cancer in general [19]. A post-transplant malignant melanoma had similar predictors as squamous cell carcinomas, but the effect of pretransplant skin cancer had a high adjusted hazard rate [19]. In one series of patients undergoing SOTR, PTM as well as a first post-transplant skin cancer carried a risk for a second skin cancer, which occurred in almost 40% of the patients. The use of Sirolimus, an inhibitor of mammalian target of Rapamycin, had a protective effect against a second malignancy, without a significant effect on graft rejection of survival. This could be related to dose reduction or discontinuation of other immune suppressing agents [20]. The susceptibility for development of cancer after organ transplantation was already high because of the use of immune suppressing agents and potential infection with oncogenic viruses [21]. Appearance of a post-transplant malignancy affected survival: a nationwide survey of SOTR showed that 13% of deaths were due to malignancy. Compared with the general population, the standard mortality ratio was 2.33. Death from lung cancer and non-Hodgkin lymphoma were most common in heart and lung recipients [27]. One monocentric series came close to the HTX-only studies, whereby only heart and lung transplants were included [18]. This study investigated the predictors for several types of post-transplant non-melanoma skin cancer. The probability of skin cancer 10 years after HTX varied between 50% and 62%. This was in the same range as lung transplant. The series confirmed that darker skin was protective for all types, while higher age was a risk factor. PTM was predictive for post-transplant basal cell carcinoma, but not for squamous cell carcinoma. The effect of PTM on mortality was not investigated.

### 4.2. Series Studying Heart Transplantation

In principle, all candidates for HTX are screened for PTM. In a large HTX series, 7.1% of the patients had PTM. Most observed PTM were lymphomas, prostate cancer, non-melanoma skin cancer and breast cancer with a median interval between cancer diagnosis and HTX of 9 years [22]. Mortality was studied in three of the four series who underwent HTX. There are several observations to be made from this analysis. The presence of PTM decreased one-year survival and increased malignancy-related mortality in one large series. Especially hematologic PTM had a large effect and seemed to be the main driver of this outcome. However, graft failure was lower in this type of patient. Cardiovascular death after HTX was also lower in this group [17]. This might be due to a lower activity of the immune system after prior cancer treatment. The prevalence of PTM has tripled in the last 20 years, making this an important observation. Advances in post-transplant cancer screening programs and immunosuppressive regimens which mitigated the risk of post-transplant cancer also could affect this result, especially for patients at high risk for developing neoplastic disease. There is a considerable variation in patient and PTM-specific characteristics such as tumor staging and treatment, which can confound the results. For survival after more than one year, the reduction in survival in patients with PTM was less obvious [17,22,24]. This might be due to improved cancer screening and treatment of patients after HTX. But a five-year follow-up might be insufficient to reach definitive conclusions. Patients with PTM had a significantly higher cancer rate of 43.8% versus 20.8%, with a 9.6% recurrence rate, but a comparable 10-year survival [22].

Post-transplant rates of cancer were investigated in three series, studying HTX [17,22,23]. The effect of diverse types of PTM on post-transplant malignancy, especially skin cancer, was investigated extensively in two HTX series [17,23]. In most series, post-transplant malignancy rates were significantly increased in patients with PTM. The susceptibility for development of cancer after organ transplantation was already high because of the use of immune suppressing agents and potential infection with oncogenic viruses [21]. The presence and the type of PTM seems to add to this effect. The recurrence rate was high in lung and breast cancer, but there was also an increase in “de novo” malignancies, i.e., a cancer different from the PTM [17]. Skin cancer (50%) was the most observed malignancy after HTX. Post-transplant lung cancer (10.9%), prostate cancer (10.0%) and lymphoproliferative disease (8.5%), were also relatively common, while other types were lower. Overall, PTM increased the risk for post-transplant malignancy by a ratio of 1.51 (1.27–1.81). Skin PTM carried a substantial risk for post-transplant skin cancer. Solid organ PTM also increased the risk for post-transplant skin cancer, but not significantly for post-transplant solid organ tumors [23]. This reassuring observation indicates that pretransplant oncologic assessment is generally accurate. The largest relative increase in post-transplant malignancy was documented for non-melanoma skin cancer. Fatalities were mostly due to highly invasive post-transplant squamous cell carcinomas, promoted by older immune suppressing agents [27]. Sun exposure also played a significant role. Another interesting aspect was the need for a second HTX in 1026 patients from the UNOS database. Patients over 60 years had a higher comorbidity rate, which included malignancy. The malignancy-related 10-year mortality was significantly higher. Age was significantly associated with 10-year mortality. The effect of PTM on the outcome was not analyzed in this specific series [28].

The type of immune suppression after HTX could also play a role in the outcome with respect to cancer rate and survival after HTX. Inhibitors of mammalian target of Rapamycin, such as Sirolimus and Everolimus, inhibit cell proliferation and angiogenesis in some types of cancers. These substances are useful as immune suppressing agents in transplant patients and as treatment for malignancies. The results, however, are not uniform. HTX patients who received Everolimus and low dose calcineurin inhibitors had better long-term renal function, but similar graft function and survival rates [29]. In another series of patients who underwent HTX, conversion to Sirolimus was associated with a lower 10-year rate of “de novo” malignancies, post-transplant lymphoproliferative disorders, and non-melanoma skin cancers. Patients without these post-transplant malignancies had better survival [30]. The 2-year cancer rate in another HTX-series showed a lower rate of post-transplant malignancy with Everolimus, compared to Mofetil Mycophenolate. This follow-up was too short to see an effect on survival [31]. Five-year cancer incidence and mortality rate after HTX were lower with mammalian target of Rapamycin inhibitors [32]. Interpretation of the results after change in regimes can be difficult because of discontinuation or dose reduction of other immune suppressants [20].

Several series indicated that patients receiving thoracic organs had an increased risk of about 50%, compared to those receiving abdominal organs [19,21]. This was, shown in series studying post-transplant skin cancer in general and for 5-year cumulative incidence of keratinocyte carcinoma. The ‘novo cancer’ death rates for abdominal versus thoracic transplants showed a comparable pattern [16]. The relative difference between transplantation of thoracic versus abdominal organs with respect to the outcome might not be an isolated feature. However, definitive conclusions should only be drawn after confirmation of these findings in future research. The current results indicate that presence of PTM affects the ‘de novo’ malignancy, recurrence of PTM and survival. This could be related to the aging population, and improvements in diagnostic approach for malignancies. Preventive strategies were proposed to decrease the risk of post-transplantation malignancy. An example is the pretransplant colorectal screening, with a detection rate of colorectal cancer below 1%. Colorectal cancer was detected in 1.2% of the patients after HTX which was lower than the 2.5% adverse event rate observed after pretransplant colonoscopy. The risk–benefit rate of pretransplant screening by endoscopy was unfavorable [33,34]. Another example is smoking, which was an independent predictor for post-transplant malignancy and mortality [35]. One could advocate for more frequent medical imaging after HTX in these patients. In areas with a high sun exposure, screening for post-transplant skin cancer could be increased in patients of white race and with a history of pretransplant skin cancer [36]. With improvements in post-transplant care and surveillance for malignancies, the risk–benefit balance seems to shift in favor of HTX and transplantation in general. For these reasons, PTM does not disqualify for HTX, but the approach should be individualized. In patients with PTM listed for HTX, collaboration with oncologists is recommended. Since PTM are diverse in type and stage, individualized risk stratification is necessary. HTX can be recommended when PTM will not impact post-transplant survival and when the risk of cancer recurrence is low. For hematologic PTM or presence of a PTM is one year or less before transplant, the risk may be higher. For early-stage PTM, treated with curative intent, the risk is low. A period of observation is specific to the given patients’ cancer history and should depend on these factors [37]. One possible example of a recommendation to offer HTX is expected 5-year cancer free survival of at least 70% [22]. Unnecessary delays for HTX should be avoided. Specific post-transplant surveillance guidelines from relevant expert societies for patients with PTM may vary over time. The corresponding recommendations for HTX candidates could need regular updates.

This study has several limitations. The outcome under study included mortality, cancer-specific mortality, and rates of specific malignancies such skin cancer. National registries can be susceptible to coding errors and missing information. This is especially true for patients with dermatologic PTM in an outpatient clinic, who failed to report this to the transplant team. Voluntary reporting of skin cancers in population-based surveys could underestimate their rate. Five of the included series studied patients after solid organ transplantation, without separate analysis of the different transplanted organs. This could be a critical issue since thoracic organ transplants might elicit a stronger immune response compared to abdominal organ transplants. However, the principles with respect to post-transplant malignancy and survival seem to be comparable for all transplanted organs. Also, detailed information about cancer type, stage, interval with HTX and treatment was absent in several series. The role of specific immune suppressing agents in the development of post-transplant malignancies was rarely assessed. Diverse regimen such as the introduction of Sirolimus, and surveillance protocols could change during the longer inclusion times and follow-up periods. The review includes post-transplantation outcome in monocentric series as well as in nationwide surveys. However, monocentric series have usually low power with an inherent risk for a type II error. Their main advantage is the detailed description of patient characteristics. Especially for skin cancers, the introduction of sun-protective behaviors could affect the outcome under scrutiny. All series were of retrospective nature with all their inherent limitations. Nevertheless, these limitations do not seem to affect the overall findings. However, the lack of information about immune suppressing agents with anticancer effects could alter the outcome after HTX and organ transplant in general. This issue needs further investigation. Furthermore, with only one author, not all requirements of the PRISMA guidelines could be followed. However, search terms, inclusion and exclusion criteria were applied and a quality assessment was performed. PubMed and Web of Science were the two sources for manuscripts. Although other literature databases are available, the most important papers were identified and included from these sources for analysis. Inclusion of one and the same patient in more than one study is limited due to differences in countries, regions, and inclusion period. In the two potentially overlapping series [17,23], the research design and outcomes under study differed substantially.

## 5. Conclusions

Pretransplant malignancy in patients with end-stage heart failure does not preclude HTX. The adverse outcome after HTX in several series was higher, compared to transplantation of abdominal organs. Current results show an increase in post-transplant malignancy, and possibly of cancer-related death, especially with respect to skin cancer. Post-transplant survival in this specific patient group is still acceptable in selected patients.

## Figures and Tables

**Table 1:** Quality assessment of the included articles by the Newcastle–Ottawa Scale.

Reference	1	2	3	4	5a	5b	6	7	8	Sum
Acuna 2018 [16]	y	y	y	y	y	n	y	y	n	7
De Rosa 2019 [18]	y	y	y	n	y	y	y	y	n	7
Garrett 2017 [19]	y	y	y	y	y	y	y	y	y	9
Karia 2016 [20]	y	y	y	y	n	y	y	y	n	7
Park 2019 [21]	y	y	y	y	y	y	y	y	n	8
Batra 2022 [17]	y	y	y	y	y	y	y	n	n	7
Youn 2022 [22]	y	y	y	y	y	y	y	y	n	8
Yoosabai 2015 [23]	y	y	y	y	y	n	y	y	n	7
Wang 2018 [24]	y	y	y	y	y	n	y	n	n	6

1: representativeness of the exposed cohort; 2: selection of the non-exposed cohort; 3: ascertainments of exposure; 4: demonstration of outcome; 5a: comparability for the most important factor; 5b: comparability: additional factors: such as demographics and other comorbid characteristics; 6: assessment of outcome: based on medical record; 7: follow-up duration; 8: adequacy of follow-up.

**Table 2:** Basic characteristics of the included series: mean age, number of HTX or SOTR and study design.

Author (Reference)	Age	Period	Region/Source	HTX	All SOTR	PTM	Design
Acuna 2018 [16]	58	1991–2010	Ontario	29	443	rate 1:2	PSM from CORR and cancer registries, with competing risk analysis
De Rosa 2019 [18]	52	1996	Australia	41	94	-	Monocentric cohort
Garrett 2017 [19]	51	2003 and 2008	US, Calif	1017	10,649	-	based on OPTN registry
Karia 2016 [20]	56	2000–2008	US, Mass	54	592	66	evaluation of immune suppression regimen in monocentric registry
Park 2019 [21]	53	1994–2012	Ontario	684	10,198	22	based on CORR registry
Batra 2022 [17]	60	2010–2020	UNOS	27,344	-	2113	based on UNOS registry
Youn 2022 [22]	58	1997–2013	R Korea	1062	-	73	monocentric
Yoosabai 2015 [23]	55	2000–2012	UNOS	23,171	-	1306	based on UNOS registry
Wang 2018 [24]	60	2004–2016	Taiwan	354	-	8	monocentric

CORR: Canadian Organ Replacement Register; HTX: heart transplantation; OPTN: Organ Procurement Transplant Network; Period: inclusion period; PSM: propensity score match; SOTR: solid organ transplant; UNOS: United Network Organ Sharing; Mass: Massachusetts; Calif: California; R: republic.

**Table 3:** Overview of the articles included with respect to the effect of PTM on outcome.

Author (Reference)	Outcome	Predictors	Result (Adjusted HR, Cum Inc. %)	*p*
Acuna 2018 [16]	all-cause mortality		1.36 (1.16–1.64)	
	cancer-specific mortality		1.85 (1.20–2.86)	
		high-risk PTM	1.81 (1.47–2.23)	
		low-risk PTM	1.06 (0.86–1.31)	
		<5 y interval	1.34 (0.73–2.47)	
		>5 y interval	2.27 (1.37–3.75)	
De Rosa 2019 [18]	probability for BCC (HTX only)		50 (33–69)% at year 10	
	for SCC (HTX only)		62 (45–78)% at year 10	
	for NMSC (HTX only)		61 (45–78)% at year 10	
	any non-melanoma skin cancer	age	1.07 (1.04–1.10)	<0.001
	(HTX and LTX)	PTM skin CA		NS
		darker skin	0.42 (0.24–0.74)	0.003
	SCC	age	1.08 (1.05–1.11)	<0.001
	(HTX and LTX)	PTM skin CA		NS
		darker skin	0.38 (0.21–0.68)	0.009
	BCC	age	1.06 (1.02–1.09)	0.001
	(HTX and LTX)	PTM skin CA	4.56 (1.67–12.42)	<0.001
		darker skin	0.49 (0.25–0.96)	0.040
Garrett 2017 [19]	skin cancer (overall)	PTM skin CA	4.69 (3.26–6.73)	<0.001
		male	1.61 (1.34–1.89)	<0.001
		white race	7.79 (5.52–11.37)	<0.001
		age > 50	2.65 (2.12–3.21)	<0.001
		thoracic TX	1.51 (1.26–1.82)	<0.001
	SCC	PTM skin CA	4.71 (3.60–6.16)	<0.001
		male	1.67 (1.41–2.01)	<0.001
		white race	8.18 (5.49–12.18)	<0.001
		age > 50	2.73 (2.24–3.34)	<0.001
		thoracic TX	1.53 (1.27–1.85)	<0.001
	malignant melanoma	PTM skin CA	7.15 (3.31–15.46)	<0.001
		male	1.26 (0.73–2.20)	0.410
		white race	7.21 (2.33–23.31)	0.010
		age > 50	1.95 (1.05–3.61)	0.030
		thoracic TX	1.33 (0.72–2.47)	0.350
Karia 2016 [20]	second skin cancer	PTM skin cancer	2.1 (1.2–3.7)	0.020
		first post skin CA	5.5 (2.5–6.4)	<0.001
		use of sirolimus	0.6 (0.4–0.9)	0.030
Park 2019 [21]	KC: SIR		6.61 (6.31–6.93)	<0.001
	Cum. inc % at 5 y (HTX)		15.04 (12.32–18.02)	<0.001
	Cum. inc % at 10 y (HTX)		26.67 (22.73–30.76)	<0.001
	KC for all SOTR	age > 65 years	9.27 (7.08–12.14)	<0.001
		white race	8.50 (4.03–17.91)	<0.001
		PTM skin cancer	4.30 (3.72–4.98)	<0.001
Batra 2022 [17]	1-y mortality	(all types PTM)	1.25 (1.09–1.44)	0.001
		(hematologic)	2.00 (1.61–2.48)	<0.001
		(breast)	1.06 (0.79–1.42)	
		(abdominal)	0.87 (0.65–1.48)	
		(other types)	1.25 (1.09–1.44)	
	Mortality > 1 and <5 year	(all types PTM)	1.00 (0.84–1.19)	0.980
		(hematologic)	1.04 (0.74–1.47)	0.480
		(breast)	0.88 (0.61–1.28)	
		(abdominal)	0.87 (0.65–1.17)	
		(other types)	1.23 (0.91–1.66)	
	Malignancy-related mortality > 1 and <5 years		18.7% vs. 10.8%	0.008
	5-y malignancy rate	(all types PTM)	1.57 (1.38–1.79)	<0.001
		(hematologic)	2.38 (1.85–3.07)	<0.001
		(breast)	1.46 (1.06–2.00)	not stated
		(abdominal)	1.17 (0.92–1.48)	not stated
		(other types)	1.69 (1.35–2.11)	not stated
Youn 2022 [22]	10-y survival as %		70.4% vs. 68.6%	0.450
	10-y freedom from non-fatal MACE as %		78.1% vs. 76.0%	0.784
	post-transplant malignancy as %		43.8% vs. 20.8%	<0.001
	post-transplant malignancy	white race	10.29 (1.19–89.07)	0.034
		BMI	1.11 (0.98–1.27)	0.109
		male	2.41 (0.67–8.67)	0.178
		age	0.97 (0.91–1.02)	0.198
	skin cancer	white race	8.88 (3.25–24.3)	<0.001
	death due to malignancy		17.2% vs. 11.6%	0.571
	PTM recurrence		9.6%	-
Yoosabai 2015 [23]	all cancers	all PTM	1.51 (1.27–1.81)	<0.010
	all cancers	skin PTM	2.38 (1.63–3.49)	<0.010
		organ PTM	1.38 (1.02–1.88)	0.040
		hematol PTM	0.95 (0.45–2.01)	0.890
		multiple PTM	2.18 (1.01–4.71)	0.050
		unknown PTM	1.38 (1.03–1.86)	0.030
	skin cancer	skin PTM	2.79 (1.82–4.28)	<0.001
		organ PTM	1.55 (1.07–2.25)	0.020
		hematol PTM	1.25 (0.55–2.83)	0.590
		multiple PTM	1.86 (0.68–5.08)	0.230
		unknown PTM	1.08 (0.71–1.64)	0.720
	organ cancer	skin PTM	1.82 (0.81–4.08)	0.150
		organ PTM	1.23 (0.69–2.19)	0.490
		hematol PTM	0.49 (0.07–3.54)	0.480
		multiple PTM	3.23 (1.01–10.38)	0.050
		unknown PTM	1.79 (1.16–2.78)	0.010
	hematologic malignancy	organ PTM	0.60 (0.09–4.28)	0.610
		unknown PTM	1.49 (0.46–4.70)	0.490
Wang 2018 [24]	5-y survival as %	with PTM	50.0 ± 17.7%	
		without PTM	68.7 ± 2.0%	
	PTM recurrence (raw data)		1 of 8 patients	

Cum inc: cumulative incidence; HR: hazard ratio; BCC + SCC: basal cell plus squamous cell carcinoma; BMI: body mass index; CA: cancer; HTX and LTX: heart and lung transplant; hematol: hematologic; MACE: major adverse cardiovascular event; KC: keratinocyte carcinoma; NMSC: non-melanoma skin cancer; PTM: pretransplant malignancy; spec mort: specific mortality; SIR: standard incidence ratio; SOTR: solid organ transplant; NS: not significant.

## Data Availability

No new data were created or analyzed in this study. Data sharing is not applicable to this article.

## Supplementary Materials

The following supporting information can be downloaded at: https://www.mdpi.com/article/10.3390/healthcare14213828/s1, File S1: PRISMA 2020 Checklist. Ref. [38] is citated in the Supplementary Material.

## Abbreviations

The following abbreviations are used in this manuscript:

BCC + SCC	Basal cell plus squamous cell carcinoma
BMI	Body mass index
CA	Cancer
Hematol.	Hematologic
HR	Hazard ratio
HTX&LTX	Heart and lung transplant
KC	Keratinocyte carcinoma
LV	Left ventricular
MACE	Major adverse cardiovascular event
NMSC	Non-melanoma skin cancer
OPTN	Organ procurement transplant network
PTM	Pretransplant malignancy
Spec mort	Specific mortality
SOTR	Solid organ transplant
